# First-Time Psychotic Symptoms in a Patient After COVID-19 Infection—A Case Report

**DOI:** 10.3389/fpsyt.2021.726059

**Published:** 2021-10-15

**Authors:** Kacper Łoś, Joanna Kulikowska, Napoleon Waszkiewicz

**Affiliations:** ^1^Department of Psychiatry, Medical University of Bialystok, Bialystok, Poland; ^2^Department of Neurology, Medical University of Bialystok, Bialystok, Poland

**Keywords:** psychotic spectrum disorders, case report, COVID-19, SARS-CoV-2, schizophrenia

## Abstract

A 39-year-old, previously healthy, white male with no personal or family history of mental illness presented with new, first-time psychotic symptoms. The new psychotic symptoms appeared on patient admission to the hospital, occurring during a diagnosis of symptomatic SARS-CoV-2 infection. On the first day of hospitalization for worsening psychotic symptoms and the appearance of aggression toward the staff, the patient was transferred to the psychiatric hospital. After the initial treatment with antipsychotics and benzodiazepines, his mental condition improved. The patient was then transferred for further treatment of his somatic condition in the internal medicine ward, with a recommendation to continue treatment in the psychiatric ward once his somatic condition was stabilized. This is one of the few reported cases of COVID-19-related psychosis in a patient without a personal or family history; moreover, this description contains important data regarding elevated IL-6, which may prove to be a key factor in the induction of new psychotic symptoms. It indicates the important need for careful monitoring of neuropsychiatric symptoms among COVID-19 patients.

## Background

The COVID-19 pandemic is caused by the SARS-CoV-2 virus. Since the beginning of the pandemic, more than 167 million people in the whole world have been infected, of which almost more than 3.5 million have died (1). The key stage of infection is a period called immune storm, wherein there is a rise in proinflammatory cytokines such as TNF-alpha, IL-1, and IL-6 (2). What is interesting from the beginning of the pandemic is that some extrapulmonary symptoms such as psychotic symptoms and features of the nervous system have been observed (3). Since the beginning of the pandemic, the possibility of nervous system involvement by the coronavirus has been raised, while psychiatric manifestations have not yet been clearly explained. An interesting phenomenon is that we have observed the occurrence of first psychotic episodes in previously mentally healthy patients. Psychotic disorders were observed in 1.4% of patients, while first psychotic episodes were observed in 0.42% (4). However, only a few such cases have been precisely described so far. In this case report, we present a patient who developed a first-time psychotic episode during hospitalization for COVID-19 with detailed description of the laboratory results including immunological changes.

## Case Presentation

A 39-year-old patient was admitted to a temporary hospital for COVID-19 patients. A positive SARS-CoV-2 RT-PCR was received on 26.04.2021. The patient had a history of fever, dyspnea, and weakness; all symptoms gradually deteriorated for 7 days. On admission, the patient was in fairly good general condition, healthy, and not taking any medications (including steroids). Numerous symmetrical crackles were heard during auscultation over the pulmonary fields. The heart rate was regular at 88 beats per minute, and blood pressure (BP) was 135/82 mm Hg. He had never received any psychiatric or drug treatment. The patient was conscious and fully oriented. He uttered delusional content, regarding the fabrication of his research. He claimed that other patients were actors and denied the existence of a pandemic. Neurological examination revealed no abnormalities or focal changes. Laboratory tests revealed elevated levels of IL-6 at 79.1 (normal to 7.0 pg/ml), C-reactive protein (CRP) at 152.8 (normal to 10.0 mg/L), and creatine kinase (CK) at 939 (normal to 200 IU/L) and slightly elevated AST, fibrinogen, D-dimers, and CK-MB. On chest computed tomography scan, lesions typical of COVID-19 (30% lung involvement) were found. The patient was prescribed levofloxacin (2 × 500 mg), ceftriaxone (1 × 2 g), dexamethasone phosphate (2 × 4 mg), remdesivir (100 mg, all intravenously, i.v.), and enoxaparin sodium (1 × 60 mg, subcutaneously, s.c.). He was treated with oxygen therapy on oxygen-conserving nasal cannula with a flow of 5 L/min, achieving a saturation of 92%. The next day, delusional content toward the staff increased. The patient was verbally and physically aggressive. He claimed that the hospital staff wanted to kill him, pulled out the intravenous drip, and refused to take any medications. The patient's family was contacted: the wife claimed that before admission to the hospital, the patient had never displayed aggressive tendencies or made similar statements. She denied that the patient had ever received psychiatric treatment or taken drugs or other intoxicating substances. After a psychiatrist was contacted, the patient, assisted by the police, was transferred to a psychiatric hospital.

During transport, the patient, despite continuous police supervision, attempted to attack the paramedic. He was admitted to the psychiatric ward with a diagnosis of acute and transient psychotic disorder. The patient was in severe anxiety, restlessness, and psychomotor agitation. He avoided eye contact, verbal contact was difficult, and he did not answer questions. Due to unpredictability and aggression, he required protection with direct coercive measures. He also refused to take medication. During hospitalization, he attacked a nurse. His temperature was 37.1°C and BP 142/85 mm Hg. In laboratory tests, CRP was elevated to 193.8 (with a normal value of 10.0). White blood cells (WBCs) were slightly elevated. Oxygen saturation was 86% with oxygen therapy of 6 L/min. In the psychiatric ward, IL-6 levels were not re-measured, due to a too short stay in the ward. The patient was fully oriented, bizarre, and non-situational and denied suicidal thoughts. An in-depth interview from the patient's wife revealed that the patient had changed mentally since admission to the temporary hospital. To date, symptoms of psychosis had never been observed. The patient's family also had no history of mental illness to date. The patient had never taken any drugs and was not addicted. He denied any alcohol abuse. Haloperidol (2 × 2.5 mg, intramuscularly, i.m.), lorazepam (1 × 2.5 mg, per os, p.o.), and olanzapine (2 x 5 mg, p.o.) were administered. Antipsychotic treatment was implemented in a psychiatric ward. The acute phase of psychosis was from admission to the temporary hospital to discharge from the psychiatric ward (4 days). The patient after being stabilized, calmed, and correctly oriented allopsychically and autopsychically, without psychotic symptoms, was discharged due to continuation of antipsychotic treatment (olanzapine 2 × 5 mg p.o.) in the internal medicine department with recommendation for further diagnostics in the psychiatric department after stabilization of somatic condition.

## Differential Diagnosis

In the absence of an alternative etiology to the development of acute psychosis, the authors concluded that the patient's symptoms were caused by SARS-CoV-2 virus infection. Possible etiologies include psychosocial stress factors in relation to the COVID-19 diagnosis or direct sequelae of infection caused by neurotropism of the virus to the central nervous system (CNS) (5). In the absence of impaired consciousness, the hypothesis of delirium was rejected.

## Discussion

Considering the novel presentation and recent history of psychotic symptoms after COVID-19 infection, the patient's diagnosis is consistent with an acute and transient psychotic disorder in the International Statistical Classification of Diseases and Health Problems (ICD)-10. Th first episode of a psychotic disorder caused by COVID-19 seems to be a worthwhile problem. There are more new information coming from various studies on the occurrence of this phenomenon. All new information as well as case reports is very valuable, considering that the pandemic is spreading around the world (6). Viral infection and its psychotic compilations have been hypothesized for years. In the past, Menninger's research confirmed influenza infection with neuropsychiatric complications (7). This raises the supposition that if the exposure to SARS-CoV-2 virus is exceptionally strong during the pandemic, this may result in neuronal inflammation in the CNS that inducts newly psychotic symptoms (8). Neurological and psychiatric diseases may be explained by the occurrence of SARS-CoV-2 infection. In a research by Taquet et al., it was revealed that within 6 months after COVID-19, a first psychotic episode occurred in 0.42% of patients. Moreover, it occurred statistically more frequently in cases associated with influenza virus or other respiratory infections (4). The mechanism by which this process may occur is the inflammatory processes of the CNS. The route of virus entry into the CNS is not yet clear (9). One possibility is direct invasion by nerve endings (for example, bulb olfactory) (10). Another mechanism is CNS damage by immune mechanisms called a cytokine storm (11). It is also worth mentioning the important role of severe stress in the induction of psychotic episodes. COVID-19 infection and fear for one's own health and life are undoubtedly strong stressors for the patient. It should be noted that steroid therapy plays a major role in COVID-19 therapy (12). The occurrence of psychiatric symptoms, such as depression and psychosis due to the use of steroid drugs, has been documented in the literature for many years. In the described case, the patient received dexamethasone in a dose of 2 × 4 mg i.v. only in the temporary hospital, when the psychiatric symptoms were already developed. It is also known that no steroids were used before admission. Taking into account the above assumption, the authors reject the hypothesis about the etiology of steroid psychosis. People should also be aware that COVID-19 infection may be a factor accelerating the onset of psychosis in predisposed people (13). All this may imply a projected rapid growth in the prevalence of psychiatric disorders in the general population after this epidemic (14).

In this case, we present a full description of the patient from the moment the first symptoms appear, with a detailed description of the laboratory results including immunological changes including IL-6. In the literature, there are studies indicating that CNS inflammation and immune dysregulation may play a role in the etiopathogenesis of schizophrenia (15–17). Nervous system inflammation can lead to white matter pathology and thus to the manifestation of schizophrenia (17). Cytokines are signaling proteins that play a critical role in the inflammatory response (18). According to a review of Dawidowski et al. (19), IL-6 is one of the most studied cytokines in schizophrenia research and there are consistent reports of elevated peripheral IL-6 levels compared to healthy populations in all patient populations (20, 21). Moreover, the authors distinguish, based on the available meta-analyses, that the only cytokine with elevated peripheral levels that can be considered as a trait marker is IL-6 (22, 23). Moreover, IL-6 increases the synthesis of acute phase proteins, and therefore, we can observe elevated CRP values (23, 24). Furthermore, it has been observed that after treatment with antipsychotics, IL-6 levels decrease (25, 26).

The patient's informed consent was obtained 7 weeks after discharge from hospital. At that time, the patient was without delusions and critical of the content of his utterances in the acute phase of psychosis. After discharge from the hospital, similar psychotic symptoms did not recur. The disadvantage of our study is the lack of long-term information about the patient and the possible result of the cerebrospinal fluid examination. Due to the enormous rate of spread of the pandemic, long-term, multi-center, case control studies are needed (27). In addition, the authors of this report postulate the need to look for a standardized panel of diagnostic tests, which, given the ubiquitous pandemic, would allow the identification of a group of people at particular risk of developing first-time psychosis spectrum disorders. Case reports and systematic literature reviews are necessary to deepen this knowledge (28). Collecting these data has the potential to enhance our mental health prevention in the context of a worldwide public health crisis (29). It is also worth noting the long-term symptoms that may result from SARS-CoV-2 infection and the impact of this virus on the CNS (30).

## Data Availability Statement

The raw data supporting the conclusions of this article will be made available by the authors, without undue reservation.

## Ethics Statement

Ethical review and approval was not required for the study on human participants in accordance with the local legislation and institutional requirements. The patients/participants provided their written informed consent to participate in this study. Written informed consent was obtained from the individual(s) for the publication of any potentially identifiable images or data included in this article.

## Author Contributions

KŁ designed the study and interpreted the data and wrote the first draft of the manuscript. KŁ and JK provided patients and relatives' data and contributed to the first draft. JK interpreted the data and wrote a revised version of the manuscript together with KŁ. NW provided constructive feedback, supervision, and a critical review of the manuscript. All authors contributed to the article and approved the submitted version.

## Funding

This study was supported by the Medical University of Bialystok, grant number SUB/1/DN/21/001/1147. The financial sponsor played no role in the design, execution, analysis, and interpretation of data.

## Conflict of Interest

The authors declare that the research was conducted in the absence of any commercial or financial relationships that could be construed as a potential conflict of interest.

## Publisher's Note

All claims expressed in this article are solely those of the authors and do not necessarily represent those of their affiliated organizations, or those of the publisher, the editors and the reviewers. Any product that may be evaluated in this article, or claim that may be made by its manufacturer, is not guaranteed or endorsed by the publisher.
